# The cost burden of oral, oral pharyngeal, and salivary gland cancers in three groups: commercial insurance, medicare, and medicaid

**DOI:** 10.1186/1758-3284-4-15

**Published:** 2012-04-26

**Authors:** Jed J Jacobson, Joel B Epstein, Frederick C Eichmiller, Teresa B Gibson, Ginger S Carls, Emily Vogtmann, Shaohung Wang, Barbara Murphy

**Affiliations:** 1 Delta Dental of Michigan, University of Michigan, 4100 Okemos Road, Okemos, MI, 48864, USA; 2 University of Illinois at Chicago, 1500 East Duarte Road, Duarte, CA, 91019-3000, USA; 3 Delta Dental of Wisconsin, 2801 Hoover Road, Stevens Point, WI, 54481, USA; 4 Thomson Reuters, 777 East Eisenhower Parkway, Ann Arbor, MI, 48108, USA; 5 Department of Epidemiology, Thomson Reuters, RPHB, 1530 3rd Avenue South, Birmingham, AL, 35294-0022, USA; 6 Formerly of Thomson Reuters, 15 Crescent Road, Lexington, MA, 02421, USA; 7 Vanderbilt University, 2220 Pierce Avenue, 777 PRB, Nashville, TN, 37232-6307, USA

**Keywords:** Oral cancer, Cost-burden, Cost of illness, Burden of illness

## Abstract

**Background:**

Head and neck cancers are of particular interest to health care providers, their patients, and those paying for health care services, because they have a high morbidity, they are extremely expensive to treat, and of the survivors only 48% return to work. Consequently the economic burden of oral cavity, oral pharyngeal, and salivary gland cancer (OC/OP/SG) must be understood. The cost of these cancers in the U.S. has not been investigated.

**Methods:**

A retrospective analysis of administrative claims data for 6,812 OC/OP/SG cancer patients was undertaken. Total annual health care spending for OC/OP/SG cancer patients was compared to similar patients without OC/OP/SG cancer using propensity score matching for enrollees in commercial insurance, Medicare, and Medicaid. Indirect costs, as measured by short term disability days were compared for employed patients.

**Results:**

Total annual health care spending for OC/OP/SG patients during the year after the index diagnosis was $79,151 for the Commercial population. Health care costs were higher for OC/OP/SG cancer patients with Commercial Insurance ($71,732, n = 3,918), Medicare ($35,890, n = 2,303) and Medicaid ($44,541, n = 585) than the comparison group (all *p* < 0.01). Commercially-insured employees with cancer (n = 281) had 44.9 more short-term disability days than comparison employees (*p* < 0.01). Multimodality treatment was twice the cost of single modality therapy. Those patients receiving all three treatments (surgery, radiation, and chemotherapy) had the highest costs of cost of care, from $96,520 in the Medicare population to $153,892 in the Commercial population.

**Conclusions:**

In the U.S., the cost of OC/OP/SG cancer is significant and may be the most costly cancer to treat in the U.S. The results of this analysis provide useful information to health care providers and decision makers in understanding the economic burden of head and neck cancer. Additionally, this cost information will greatly assist in determining the cost-effectiveness of new technologies and early detection systems. Earlier identification of cancers by patients and providers may potentially decrease health care costs, morbidity and mortality.

## Background

Health care costs for patients; employers, and health care plans are rising in the U.S. [1,2]. Thus, methods for decreasing health care expenditures are being rigorously scrutinized. Cancer diagnosis and treatment contributes significantly to these costs [3-6]. Head and neck cancers are of particular interest because they are extremely expensive to treat, have a high morbidity, and of those individuals that survive only 48% return to work [7]. Further, recent evidence suggests that patients with cancer are at a particular risk for bankruptcy [8]. To minimize cost and risk, employers are grappling with enacting programs such as banning smoking on the work property, supporting smoking cessation programs, and making health care benefit decisions on new early detection and screening tools [9].

Head and neck cancers are a group of tumors that arise from five primary sites (larynx, pharynx, oral cavity, salivary glands and paranasal sinuses). Cancers arising from three of the sites or subsites (oral cavity, oral pharynx and salivary gland tumors – OC/OP/SG) present with symptoms that may prompt a visit to an oral health provider, thus these cancers are the focus of this analysis. Known risk factors for oral and oropharyngeal cancers include cigarette smoking, chewing tobacco and alcohol consumption [10]. Human Papilloma Virus (HPV) has recently been implicated as a causal factor for oropharyngeal cancers and now accounts for 40 to 60% of cases [10-12]. HPV associated tumors tend to occur in younger patients lacking the traditional risk factors of tobacco and alcohol use. The incidence of oropharynx cancers is rising rapidly [13] as is the incidence of oral tongue lesions in younger adults and women [14]. Salivary gland tumors are a relatively rare group of tumors that behave in a heterogeneous manner; and although they are uncommon, they result in substantial morbidity.

Treatment for individuals with OC/OP/SG cancers includes surgery, radiation therapy and chemotherapy [15]. Each treatment modality is associated with distinct acute and late treatment effects. With advances in surgical techniques, particularly in the field of reconstruction, the functional and cosmetic outcomes for individuals undergoing primary surgical resection may be excellent, even for those patients with large tumors. However, cancer surgery can still result in substantial functional impairment and disfigurement. Radiation therapy is also associated with severe acute and late affects. Common acute toxicities include xerostomia, painful mucositis, dermatitis, and severe dysphagia which may require feeding tube placement. Late radiation effects include neck and shoulder fibrosis and edema, trismus, mucosal sensitivity, late effect xerostomia and dental caries with associated dental loss [16]. When used in combination with chemotherapy, these effects may be exacerbated. It should be noted that head and neck cancer is associated with a high rate of mood disorders including anxiety and depression and higher rates of depression have been observed in individuals with tumor and treatment related disfigurement [17].

In general, the more advanced stage the cancer at diagnosis, the worse the prognosis. For example, the overall five-year survival rate is approximately 60% for all stages of oral cancer patients in the U.S., but survival increases to 83% when the cancer is detected in its early stage [18]. Unfortunately, most patients are diagnosed with locally advanced disease. Only 36% - 41% of oral cavity/oral pharyngeal cancers are detected early [18]. In addition to improving survival, early detection of OC/OP/SG cancers may identify early stage disease which requires less aggressive and less toxic therapies. Thus, for OC/OP/SG cancers screening and early detection have become an important focus for health care providers. New early detection and screening tools are being developed in the hopes of increasing the rate of early cancer diagnosis [9]. By including screening as part of regular dental exams, dentists and hygienists have the opportunity to detect these cancers early, decreasing morbidity and mortality. Additionally, educational programs to raise awareness among health care providers and programs instructing individuals on self-examination may result in earlier detection and greatly reduce the high cost and mortality of OC/OP/SG cancers.

It may be hypothesized that early detection of cancers may diminish the cost of care for individuals and employers, thus providing additional impetus for more effective screening efforts. Despite the prevalence of OC/OP/SG cancers and the potential for disability and disfigurement that may result from treatment, previous research on the direct and indirect cost burden is limited. Most of the research has focused on the broader category of head and neck cancers [3,5,19]. To our knowledge, a comprehensive study of the direct and indirect cost burden of these cancers among relatively large samples of Medicare, Medicaid and commercially insured patients has not been conducted. To fill this gap in knowledge, we conducted a study to determine the direct costs of OC/OP/SG cancers during the first year after diagnosis. We examined three cohorts of individuals: Commercially-insured enrollees, enrollees with Medicare and supplemental benefits from their employer, and Medicaid beneficiaries. For employees within the Commercially-insured sample, we calculated the indirect costs of oral or pharyngeal cancer as measured by short term disability costs. We also estimated spending by treatment modality (e.g., surgery, radiation or combined treatment) and examined patterns in spending over time from six months to three years after the oral cavity, oral pharynx and salivary gland (OC/OP/SG) cancer diagnosis.

## Methods

### Study design

We conducted a retrospective, observational study of the direct and indirect cost burden of OC/OP/SG cancer using the health care experiences of employees and their dependents with employer- sponsored health insurance (Commercial), retirees and their dependents with Medicare Supplemental coverage from their former employer, and Medicaid beneficiaries in 11 states. To calculate the cost burden of illness within each payer, a comparison group of individuals without OC/OP/SG cancer was created using propensity scoring techniques matching people based on their socio-demographic characteristics (e.g., plan type, year of diagnosis) and health status. The cost burden of illness for OC/OP/SG cancer was then estimated by comparing spending for cases with OC/OP/SG cancer to spending in the matched comparison group.

### Data sources

Data for this study were obtained from the 2004–2008 Thomson Reuters *MarketScan®* Databases: Commercial Claims and Encounters Database (CCAE), Medicare Supplemental and Coordination of Benefits Database, Medicaid Multi-State Database and the Health Productivity and Management (HPM) Database.

The CCAE database contains the enrollment and health care (medical and drug) claims experience of several million employees and their dependents that are covered annually under a variety of health plans offered by medium-sized and large firms. The MarketScan Medicare Supplemental Database contains the enrollment and health care claims of millions of individuals with Medicare supplemental insurance paid for by employers. Both the Medicare-covered portion of payment (represented as Coordination of Benefits Amount, or COB) and the employer-paid portion are included in this database. The MarketScan Medicaid Multi-State Database comprises data from 11 contributing geographically disperse states and contains the administrative claims experience of millions of Medicaid enrollees (e.g., over 5 million enrollees in 2007). The CCAE, Medicare Supplemental, and Medicaid databases all include inpatient, outpatient, emergency room and outpatient prescription drug claims, linked by a unique patient identifier, except for outpatient prescription drugs in the Medicaid database for which there is no data. The Medicaid database has all claims, except for outpatient prescription drug claims for the small share of enrollees who were dually eligible for Medicare (6% of Medicaid sample with OC/OP/SG cancer). For Commercially insured patients, switching from one health plan to another within a single employer did not constitute disenrollment. Data from all carve-out plans (e.g., prescription drug, mental health) were also included in the database. Finally, the MarketScan HPM Database is linkable via a unique enrollee identifier to the medical and pharmacy experience of a subset of employees in the CCAE database whose employers contribute their short-term disability claims experience.

The data conformed to the Health Insurance Portability and Accountability Act of 1996 (HIPAA) confidentiality requirements, so neither informed consent nor Institutional Review Board (IRB) approval were necessary for this study.

### Patients with oral cavity, oral pharynx or salivary gland cancers

OC/OP/SG cancer patients were identified via at least one inpatient claim with an International Classification of Disease, 9^th^ Revision, Clinical Modification (ICD-9-CM) code for oral cavity, oral pharynx, or salivary gland cancer: ICD-9-CM codes 141.0-141.9 (base of tongue), 142.0-142.9 (major salivary gland), 143.0-143.9 (gum), 144.0-144.9 (floor of mouth), 145.0-145.9 (other sites of mouth), 146.0-146.9 (oropharynx), 149.0-149.9 (other lip, oral cavity and pharynx) or two outpatient claims with an ICD-9-CM code for OC/OP cancer for an office visit and/or emergency department visit that were at least 30 days apart. The second outpatient claim was required to confirm the first diagnosis and ensure that patients had active disease, as reflected by utilization of medical services. The service date of the first observed claim with a diagnosis of OC/OP/SG cancer must have occurred between 2005 and 2007 and this date was designated as the index date. Patients must have been continuously enrolled for at least 6 months before and 6 months after the index date. However, the primary analysis only considered patients with at least 1 year of follow-up after the index date. A secondary analysis examined results for cohorts of patients with varying lengths of follow-up (from 6 months to 3 years). Only patients age 18 and older were included in the study.

The focus of the study was to track cost for patients at the start of a new episode of care for an OC/OP/SG cancer. Patients were excluded if they had a diagnosis of OC/OP/SG cancer any time in the 6 month pre-index period. A total of 3,918 patients in the CCAE, 2,306 patients in the Medicare Supplemental and 588 patients in the Medicaid database met the criteria for inclusion in this study. Three patients from the Medicare Supplemental Database and three from the Medicaid Database were excluded because they were extreme outliers compared to the rest of OC/OP/SG cancer patients (>$800,000 total costs for Medicare And > $1,000,000 for Medicaid).

Companies supplying short-term disability data in addition to medical claims information were a subset of those contributing medical claims data. Short-term disability information was collected only for employees and was not available for spouses or dependents. Of the Commercially-insured OC/OP/SG cancer patients, 281 had short-term disability information.

### Comparison group and propensity score matching

We created matched comparison groups for the primary cohort of OC/OP/SG cancer patients who could be followed for at least 1 year after their index diagnosis. Separate comparison groups were created for each payer group (Commercial, Medicare Supplemental, and Medicaid). To construct the comparison group, all enrollees who were at least 18 years old and had at least 18 months of continuous medical and prescription drug enrollment with none of the ICD-9-CM codes listed above within each database were selected. The enrollees meeting these criteria were randomly assigned an index date so the distribution of index dates matched the distribution of index dates for those with OC/OP/SG cancer. All comparison group members must have been continuously enrolled for 6 months before and 1 year after their assigned index date to match the enrollment requirements for the main analysis of 1 year post- index costs.

A propensity score was estimated for each OC/OP/SG cancer patient and comparison group member using logistic regression models to predict the likelihood of having OC/OP/SG cancer, as a function of socio-demographic characteristics, plan type, health status and year of index date (described below). Separate regressions were estimated for each data source (Commercial, Medicare, and HPM). For each payer sample, we matched by subgroups based on the site of the index OC/OP/SG cancer diagnosis. The predicted value of the dependent variable from the logistic regression (i.e., the predicted probability of having OC/OP/SG cancer) was the propensity score assigned to each patient.

One-to-one matching of comparison patients to employees with cancer was then performed within each of the groups, based on the value of the propensity score, using a greedy matching algorithm [20]. This produced matched sets of patients and comparison group members chosen from a large population who had similar demographic, plan type, location, and health status characteristics.

### Patient characteristics

We matched on the following socio-demographic variables that are well-known determinants of health care utilization and expenditures: age, gender, U.S. Census region and whether the enrollee’s residence was in an urban or rural area. In addition, we also matched on some employee characteristics available for the CCAE and Medicare samples: relationship (employee or spouse/child/dependent) and classification (active full time vs. other). Median income and percent of college graduates (among residents aged 25 years and older) of the enrollee’s ZIP code from the 2000 Census were included as a proxy for socio-economic status.

The matching regression also included insurance plan type. For Medicare and CCAE, the following plan types were included in the sample: indemnity plan, exclusive provider organization/point of service plan (EPO/POS), preferred provider organization (PPO), health maintenance organization (HMO), or capitated point of service plan (Capitated POS). Data on health plan type were missing for some employees and this was accounted for in the statistical models. The matching regression also controlled for Medicaid eligibility category and an indicator for dual eligibility for Medicaid and Medicare.

Pre-diagnosis health status was measured by the Charlson Comorbidity Index (CCI), a numeric scale reflecting the risk of death or serious disability in the next year based on the presence of a diagnosis for one of 19 conditions in the 6 month pre-period [21]. We created another measure of health status by modifying the CCI to exclude cancer diagnoses and other diagnoses related to risk factors for OC/OP/SG cancers (excluded any malignancy, metastatic solid tumor and chronic pulmonary disease). By using the modified CCI for matching, we were able to obtain a matched comparison group that had a similar number of other comorbidities as patients with OC/OP/SG cancer. Since the CCI does not encompass mental health comorbidities, a count of the number of Psychiatric Diagnosis Groups (PDGs) contained in the patient’s claims history in the 6 month pre-period was also included. There are 11 PDGs, representing the mental health burden of each enrollee [22]. Examples include organic mental disorders, substance use disorders, depression, and bipolar disorder.

The year of the index date was also included in the propensity score equation to represent trends in diagnosis, treatment, or medical spending.

### Outcome variables

#### Expenditures

The primary outcomes for this study were direct medical expenditures and indirect (short-term disability) expenditures during the year following the index date. Direct health care expenditures were measured as allowed charges for inpatient admissions, outpatient hospital visits, office visits, emergency department visits, and outpatient prescription drugs. All sources of payment were included in the measure of health care expenditures, including payments from the employer, plan, patient (e.g. copayments and deductible), Medicare, if eligible, and any coordination of benefits (other payers) payments. All dollar metrics were adjusted to year 2009 values using the Consumer Price Index Inflation Calculator from the Bureau of Labor Statistics [23].

#### Short term disability

Short-term disability (STD) was recorded as the number of days with short term disability benefits. These hours were converted to costs using an hourly rate of $29.37 representing compensation from civilian workers. This category of civilian workers includes private industry employees in addition to state and local workers. Since STD benefits typically do not replace full wages, the value for each STD day was set at a level of 70% compensation [24]. For example, an enrollee with 5 days of STD would have STD costs of 5 days*70% compensation*$29.37 per hour*8 h per day = $822.36.

### Analysis of spending patterns

#### Cost burden of illness for oral cavity or oral pharynx or salivary gland cancer

Descriptive statistics were calculated for patients with OC/OP/SG cancer and their matched comparison group within each payer group. Differences were tested using student’s t-tests for continuous variables and Pearson chi-square tests for categorical variables. The cost burden of OC/OP/SG cancer was calculated as the difference in direct or indirect expenditures between cases with OC/OP/SG cancer and their matched comparison group during the year after the index date. Student’s t-tests were used to assess the statistical significance of these differences.

### Spending by treatment modality

To proxy for cancer staging (severity), treatment modality (surgery only, radiation only, chemotherapy only, the combinations the three modalities or no known treatment) was measured by assessing each patient’s claims for specific HCPCS, CPT-4 and ICD-9-CM procedure codes as well as drug codes for chemotherapy (any antineoplastic drugs) in the year after the index date (Additional file 1).

#### Time trends in spending

Since the cause of disenrollment was not observed and mortality rates are non-negligible, we formed cohorts with varying lengths of time of enrollment to determine the costs of survivorship within the plan. To do so, four cohorts of cancer patients were created based on the length of enrollment. Each cohort could be followed over different time frames:

· (6 month cohort) enrolled at least 6 months after the index date but less than 12 months,

· (1 year cohort) enrolled at least 12 months after the index date but less than 24 months,

· (2 year cohort) enrolled at least 24 months after the index date but less than 36 months,

· (3 year cohort) enrolled at least 36 months after the index date.

Direct costs in 6 month intervals were created for each cohort of patients (6 month, 1 year, 2 year and 3 year as enrollment allowed) and plotted in a graph. SAS 9.2 was used for all analyses and a two- sided alpha of 0.05 was considered statistically significant.

## Results

### Patient sociodemographics

The Commercial and Medicare samples were drawn from similar populations (employees and their dependents in self-insured plans), and they were largely similar except for age and retirement status. In the Commercial sample, individuals with OC/OP/SG cancer (N = 3,918) were on average 53.4 years old and 68.7% were male (Table 1). Medicare patients were older (average age 74.5 years) and the sample included more men than women (65.4% male). Individuals with OC/OP/SG cancer in the Commercial and Medicare samples were more likely to live in urban areas (81.7% Commercial, 81.3% Medicare). Approximately half of the Commercially insured OC/OP/SG cancer individuals were active, full time employees and more than half had health care coverage under a preferred provider organization (56.0%). The Medicare sample included retirees who were no longer working and 61.4% were enrolled in a comprehensive insurance plan type. After propensity score matching, characteristics of individuals with OC/OP/SG cancer were similar to the matched comparison group. The Medicaid sample was smaller than the other samples and included 585 patients with OC/OP/SG cancer who were on average 53.4 years old. The Medicaid sample had a higher burden of comorbid conditions, as indicated by a higher Charlson Comorbidity Index and a larger number of Psychiatric Diagnosis Groups.

**Table 1:** Characteristics of patients with OC/OP/SG cancer and matched comparison group

	**Commercial Database**			**Medicare Supplemental Database**			**Medicaid Database**		
	**OC/OP/SG Cancer**	**Matched Comparison group**	**P-Value**^**A**^	**OC/OP/SG Cancer**	**Matched Comparison group**	**P-Value**^**A**^	**OC/OP/SG Cancer**	**Matched Comparison group**	**P-Value**^**A**^
Number of patients	3,918	3,918		2,303	2,303		585	585	
Age in Years	53.42	52.77	<0.001	74.51	74.34	0.392	53.36	52.52	0.148
Age Group									
18-34	3.2	3.6	0.643	0.0	0.0	0.673	3.8	4.8	0.776
35-44	8.9	8.8		0.0	0.0		9.9	8.7	
45-54	34.9	34.1		0.2	0.4		36.6	37.9	
55-64	53.0	53.5		1.2	1.4		43.8	43.6	
65-74	0.0	0.0		52.3	51.8		4.3	3.1	
75-84	0.0	0.0		37.6	37.7		1.5	1.2	
85+	0.0	0.0		8.7	8.7		0.2	0.7	
Gender									
Male	68.7	69.3	0.574	65.4	64.6	0.557	58.8	58.1	0.813
Female	31.3	30.7			34.6	35.4	41.2	41.9	
Urbanicity									
Urban	81.7	82.5	0.471	81.3	82.6	0.489	67.2	67.5	0.604
Rural	17.7	17.1		18.4	17.1		32.6	32.5	
Missing	0.6	0.4		0.3	0.3		0.2	0	
Geographic Region									
Northeast	12.1	12.5	0.392	9.6	9.7	0.191	n.a		
Northcentral	26.1	27.6		38.2	41.5				
South	44.6	43.8		32.8	31.0				
West	16.6	15.6		19.1	17.5				
Unknown	0.6	0.5		0.3	0.3				
Employee Relationship									
Employee	69.5	69.6	0.006	78.6	79.5	0.208	n.a		
Spouse	29.8	28.9		21.2	20.4				
Child/dependent	0.7	1.5		0.2	0.0				
Employee Classification Salaried/Hourly									
Salary	19.4	20.8	0.003	23.4	23.8		n.a		
Hourly	23.3	25.7		38.2	37.9				
Unknown/missing	57.4	53.5		38.4	38.4				
Union/non-union									
Union	22.3	25.6	<0.001	40.9	40.3	0.278	n.a		
Non-union	33.4	34.7		34.7	36.8				
Unknown/missing	44.3	39.7			24.4	22.9			
Employment									
Active, full time	50.1	50.1	<0.001	1.5	1.4	0.131	n.a		
Active, part time/seasonal	0.9	0.8		0.0	0.0				
Retire	23.4	27.2		90.7	88.9				
Other/unknown/missing	25.7	21.9		7.8	9.6				
Insurance Plan Type									
Comprehensive/FFS	11.6	11.5	0.522	61.4	62.7	0.540	64.4	63.2	0.671
EPO	0.6	0.5		0.0	0.0		35.6	36.8	
HMO/Managed Care	16.3	15.8		7.7	8.0				
POS	11.2	11.6		1.9	1.6				
PPO	56.0	55.9		27.5	25.7				
POS with capitation	1.3	1.7		0.0	0.0				
CDHP	1.9	2.1		0.1	0.3				
Missing	1.2	0.9		1.4	1.7				
Year of Index Date									
2005	40.6	42.7	0.155	45.5	47.7	0.225	54.5	56.1	0.739
2006	32.6	31.1		30.0	29.8		26.7	26.8	
2007	26.8	26.2		24.4	22.5		18.8	17.1	
Charlson Comorbidity Index (CCI)	1.67	0.31	<0.001	1.88	0.73	<0.001	2.31	0.81	<0.001
Modified CCI^B^	0.21	0.20	0.2457	0.47	0.43	0.1303	0.59	0.56	0.6345
Number of psychiatric	0.12	0.11	0.414	0.06	0.05	0.128	0.50	0.53	0.633
Diagnosis Groups (PDGs)									
Median Household Income in ZIP	$48,710	$48,799	0.828	$46,118	$46,708	0.128	$33,305	$32,782	0.405
Fraction College Graduates in ZIP	0.26	0.26		0.23	0.24		0.16	0.16	0.834
Race/Ethnicity	n.a			n.a					
White							52.3	49.4	0.562
Black							34.7	36.2	
Hispanic							0.7	14.0	
Other							12.3	14.0	
Medicaid Eligibility	n.a			n.a					
Blinded/Disabled							82.1	82.4	0.957
Aged							4.3	3.9	
Other							13.7	13.7	
Medicare Eligibility	n.a			n.a					
Yes							6.0	5.5	0.706
No							94.0	94.5	

For sample with 1 year of follow-up in the Medical care data.

Notes

A. P-value from test of the significance of difference between the OC/OP cancer group and matched comparison group.

B. Modified CCI recalculates the CCI excluding tobacco and cancer related diagnoses.

### Cost burden of oral cavity, oral pharynx, or salivary gland cancer

**Figure 1:**
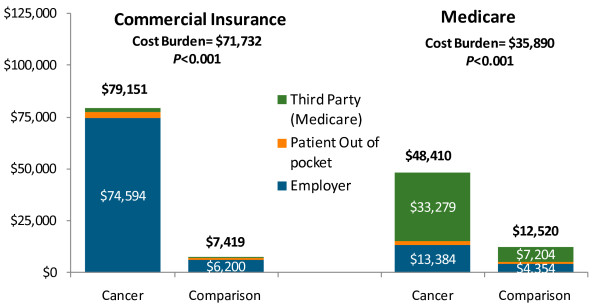
**Total costs by payer during year after oral/pharyngeal cancer diagnosis. Notes: For sample with 1 year of follow-up.** Medical and drug expenditures were adjusted to 2009 dollars using Medical Care CPI. The Cost Burden is calculated as the difference between the mean values for the OC/OP/SG cancer and matched comparison group. The p-value is from a test of statistically significant difference between the OC/OP/SG cancer and comparison group.

For the Commercial sample, total annual health care spending during the year after the index diagnosis was $79,151 (std.dev. $86,170) for individuals with OC/OP/SG cancer, compared with $7,419 (std.dev. $22,665) for the comparison group; the difference of $71,732 (*p* < 0.001) representing the cost burden of oral cancer (Table 2 and Figure 1). Total annual health care spending in the Medicare Supplemental sample was $48,410 (std.dev. $61,599), which was $35,890 higher than the comparison group (*p* < 0.001).

**Table 2:** Mean payments, standard deviation, and cost burden, by payer and population

**OC/OP/SG Cancer**		**Matched Comparison Group**	**Cost Burden**^**A**^	**Cost Burden P-value**^**B**^
COMMERCIAL^C^				
Number of enrollees	3,918	3,918		
Total Payments, $	79,151	7,419	71,732	<0.001
	(6,170)	(22,665)		
Employer Payments, $	74,594	6,200	68,394	<0.001
	(85,023)	(20,046)		
Out of pocket payments, $	2,962	829	2,133	<0.001
	(4,990)	(1,211)		
Third Party Payments,$	1,595	390	1,205	<0.001
	(12,915)	(6,160)		
MEDICARE^D^				
Number of enrollees	2,303	2,303		
Total Payments, $	48,410	12,520	35,890	<0.001
	(61,599)	(27,947)		
Employer Payments, $	13,384	4,354	9,030	<0.001
	(23,742)	(12,513)		
Out of pocket payments, $	1,747	962	785	<0.001
	(3,068)	(966)		
Medicare and Third Party Payments, $^E^	33,279	7,204	26,075	<0.001
	(53,223)	(23,036)		
MEDICAID^F^				
Number of enrollees	585	585		
Total Payments, $	59,404	14,863	44,541	<0.001
	(74,919)	(28,432)		

Standard deviation in parentheses.

For sample with 1 year of follow-up.

Medical and drug expenditures were adjusted to 2009 dollars using Medical Care CPI.

Notes

A: The Cost Burden is calculated as the difference between the mean values for the OC/OP cancer and matched comparison group.

B: The p-value is from a test of statistically significant difference between the OC/OP cancer and comparison group.

C: 6 Patients with a total medical costs exceeding $800,000 in the Commercial database were flagged as outliers, and were excluded before the matching.

D: 9 Patients with a total medical costs exceeding $800,000 in the Medicare database were flagged as outliers, and were excluded before the matching.

E: In the Medicare sample, third party payments or coordination of benefits are primarily Medicare payments, since Medicare pays first of all of the insurance plans that cover an employee. In the Commercial sample, third party payments may come from a variety of sources, such as benefits from a spouse.

F: 3 Patients with total medical costs exceeding $1,000,000 in the Medicaid database were flagged as outliers, and were excluded before the matching.

**Figure 2:**
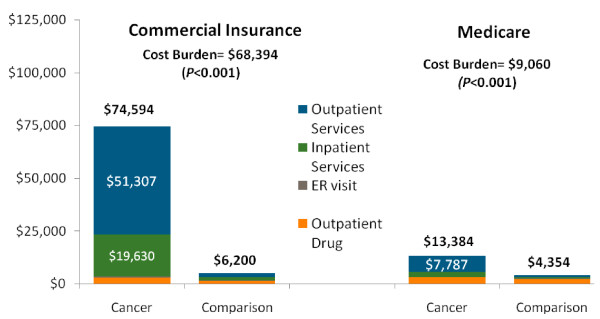
**Employer costs by service type, for Commercial Insurance and Medicare Enrollees.** Notes: For sample with 1 year of follow-up. Medical and drug expenditures were adjusted to 2009 dollars using Medical Care CPI. The Cost Burden is calculated as the difference between the mean values for the OC/OP/SG cancer and matched comparison group. The p-value is from a test of statistically significant difference between the OC/OP/SG cancer and comparison group.

Employers on average paid 94% ($74,594, std. dev. $85,023) of the total annual health care spending for individuals with OC/OP/SG cancer enrolled in Commercial plans (Figure 2). For these individuals, the employer share of the cost burden of OC/OP/SG cancer was $68,394 (*p* < 0.001). In the Medicare Supplemental sample, Medicare is the primary payer. Consequently, the employer’s share of total health care costs was substantially lower, although still sizeable ($13,384, std. dev. $23,742), representing about 28% of total health care costs. The employer’s share of the cost burden in the Medicare Supplemental sample was $9,030 (*p* < 0.001). Outpatient care accounted for the largest share of employer costs for both the Commercial sample ($51,307 or 68.8% of employer costs) and Medicare sample ($7,757 or 58.2% of employer costs), shown in Figure 2. Total annual health care expenditures in the Medicaid sample was $59,404 (std. dev. $74,919) for patients with OC/OP/SG cancer, which was $44,541 higher than the matched comparison group.

### Treatment modality and health status

Multiple modalities of treatment were more common than single modality treatment (Table 3). Forty-five percent of the Commercial sample received multimodal therapy, 30.4% receive single modality therapy, and 24.5% of patients had unknown therapy (did not have claims for any of the therapies). In Medicare, 40.6% had multimodal therapy, 36.6% had single modality therapy, and 22.8% had unknown therapy. In the Commercial population, the most common combined therapy was surgery plus either radiation (17.1%) or surgery plus radiation and chemotherapy (17.4%). In the Medicare population, surgery plus radiation (18.8%) was the most frequent multiple modality therapy, followed by surgery plus radiation and chemotherapy (12.2%). For patients receiving single modality therapy, surgery predominated in both the commercial (22.5%) and Medicare (28.7%) populations. About one third (34.9%) of Medicaid patients did not receive known therapy and the most common therapy was surgery (15.9%), followed by radiation combined with chemotherapy (12.0%) and surgery combined with both radiation and chemotherapy (11.6%).

**Table 3:** Mean Health Status by treatment modality

		**Known Treatment Modalities**									
	**Total OC/OP/SG Cancer**	**Radiation**	**Surgery**	**Chemotherapy**	**Radiation + Chemotherapy**	**Surgery + Chemotherapy**	**Surgery + Radiation**	**Surgery + Radiation + Chemotherapy**	**Total known modalities**^**A**^	**Unknown**	**P- value**^**B**^
**COMMERCIAL**											
Number of Patients	3918	233	882	77	361	55	670	681	2959	959	
Percent of oral cancer group	100.0%	5.9%	22.5%	2.0%	9.2%	1.4%	17.1%	17.4%	75.5%	24.5%	
Charlson Comorbidity	1.67	2.54	0.80	3.40	3.15	2.20	1.80	2.19	1.86	1.06	<0.001
Index (CCI)											
Modified CCI^C^	0.21	0.29	0.21	0.43	0.24	0.25	0.27	0.18	0.23	0.15	<0.001
Number of Psychiatric Diagnosis Groups	0.12	0.12	0.12	0.21	0.15	0.15	0.12	0.11	0.12	0.11	<0.001
**MEDICARE**											
Number of Patients	2303	130	660	53	150	69	434	281	1777	526	
Percent of oral cancer group	100.0%	5.6%	28.7%	2.3%	6.5%	3.0%	18.8%	12.2%	77.2%	22.8%	
Charlson Comorbidity Index (CCI)	1.88	2.88	1.32	2.45	2.88	2.33	2.13	2.35	2.00	1.49	<0.001
Modified CCI^C^	0.47	0.53	0.40	0.36	0.52	0.49	0.58	0.54	0.49	0.41	<0.001
Number of Psychiatric	0.06	0.12	0.06	0.11	0.07	0.04	0.04	0.07	0.06	0.07	<0.001
Diagnosis Groups											
**MEDICAID**											
Number of Patients	585	52	93	29	70	7	62	68	381	204	
Percent of oral cancer group	100.0%	8.9%	15.9%	5.0%	12.0%	1.2%	10.6%	11.6%	65.1%	34.9%	
Charlson Comorbidity	2.31	3.15	1.63	3.34	3.20	2.86	1.95	2.18	2.43	2.07	<0.001
Index (CCI)											
Modified CCI^C^	0.59	0.50	0.70	0.62	0.80	0.57	0.82	0.62	0.69	0.41	<0.001
Number of Psychiatric Diagnosis Groups	0.50	0.62	0.56	0.31	0.59	0.86	0.50	0.54	0.55	0.43	0.536

A. Total known modalities is the sum of previous 7 columns (radiation through surgery + radiation + chemotherapy.

B. P-value is from chi square test for a significant difference between the treatment modalities (including unknown modality).

C. Modified CCI excludes cancer and tobacco related diagnoses from the CCI.

The pre-diagnosis Charlson Comorbidity Index (CCI), the Modified CCI (cancer and tobacco related conditions have been removed), and count of Psychiatric Diagnosis Groups (PDGs) varied with treatment modalities (all *p* < 0.001), except for the number of PDGs in the Medicaid sample (*p* = 0.536). In the Commercial sample, the chemotherapy group had the highest average CCI (3.40), Modified CCI (0.43) and highest count of PDGs (0.21). Radiation and combined radiation and therapy groups had the highest CCI in the Medicare sample (2.88) and a relatively high modified CCI (0.53 and 0.52), but not the highest Modified CCI in the Medicare sample (0.58, combined surgery and radiation).

### Costs by treatment modality

**Figure 3:**
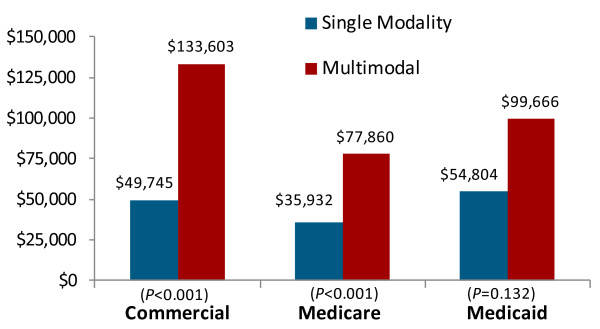
**Health care costs of single versus multimodal cancer treatment.** Notes: For sample with 1 year of follow-up. Medical and drug expenditures were adjusted to 2009 dollars using Medical Care CPI. The p-value is from a test of statistically significant differences between OC/OP/SG cancer patients who were treated with a single modality and those treated with multiple modalities.

Figure 3 demonstrates the higher average cost of late-stage multiple modality cancers compared to early stage single modality treatment. Although information on stage was not available, the use of single modality therapy was used as a surrogate indicator of early stage disease and combined modality treatment was used to represent patients with more advanced disease based upon the general association between severity and the use of more aggressive multimodal therapies [25]. Regardless of the type of insurance, multimodal therapy cancers were about double the cost of single-therapy cancers (*p* < 0.001 for Commercial and Medicare, *p* = 0.132 for Medicaid). Those receiving all three treatments (surgery, radiation, and chemotherapy) had the highest cost of care, ranging from $96,520 in the Medicare population to $153,892 in the Commercial population (Table 4). The highest cost single modality therapy was radiation alone at $66,670 in the Medicare population and $105,422 in the Commercial population.

**Table 4:** Mean and standard deviation of payments, by treatment modality

	**TOTAL OC/OP/SG Cancer**	**Known Treatment Modalities**								**Unknown**	**P-value**^**B**^
		**Radiation**	**Surgery**	**Chemotherapy**	**Radiation + Chemotherapy**	**Surgery + Chemotherapy**	**Surgery + Radiation**	**Surgery + Radiation + Chemotherapy**	**TOTAL Known treatment modalities**		
COMMERCIAL											
Number of enrollees	3,918	233	882	77	361	55	670	681	2959	959	
Total $	79,151	105,422	32,476	79,072	137,315	137,277	110,679	153,892	99,822	15,373	<0.001
	(86,170)	(82,487)	(47,082)	(72,498)	(77,676)	(125,408)	(75,189)	(92,759)	(88,715)	(25,828)	
Rx Drug $	3,541	3,900	2,207	7,858	5,637	8,862	3,602	5,079	4,006	2,107	<0.001
	(5,487)	(4,484)	(3,725)	(11,149)	(6,560)	(14,613)	(4,883)	(6,628)	(5,996)	(3,061)	
ER $	723	1,614	330	637	1,641	495	1,035	805	871	265	<0.001
	(4,282)	(6,465)	(1,679)	(1,624)	(9,247)	(1,127)	(5,063)	(3,571)	(4,854)	(1,403)	
Inpatient $	20,691	18,778	15,950	20,623	24,166	51,659	28,778	38,334	26,017	4,257	<0.001
	(44,150)	(43,736)	(38,519)	(31,899)	(43,557)	(95,505)	(44,889)	(60,405)	(48,933)	(14,789)	
Outpatient $	54,197	81,130	13,989	49,954	105,871	76,260	77,264	109,674	68,927	8,745	<0.001
	(60,854)	(61,492)	(16,468)	(52,256)	(58,144)	(84,987)	(54,602)	(57,678)	(62,582)	(17,619)	
MEDICARE											
Number of enrollees	2,303	130	660	53	150	69	434	281	1777	526	
Total $	48,410	66,670	28,138	57,590	85,462	53,526	67,020	96,520	57,969	16,113	<0.001
	(61,599)	(59,605)	(47,551)	(72,809)	(72,710)	(48,819)	(61,044)	(79,430)	(65,926)	(24,099)	
Rx Drug $	3,798	4,362	3,232	6,056	4,332	6,400	3,622	5,102	4,006	3,096	<0.001
	(4,558)	(5,284)	(3,249)	(5,723)	(4,428)	(6,749)	(3,918)	(4,932)	(4,323)	(5,220)	
ER $	259	396	175	729	461	294	232	423	289	157	<0.001
	(898)	(966)	(453)	(1,868)	(983)	(574)	(1,270)	(1,160)	(981)	(517)	
Inpatient $	12,863	10,860	12,240	15,576	15,527	12,597	17,417	20,164	15,047	5,483	<0.001
	(29,872)	(24,140)	(31,434)	(42,028)	(34,848)	(16,582)	(32,743)	(35,304)	(32,264)	(17,905)	
Outpatient $	31,490	51,051	12,492	35,230	65,141	34,235	45,750	70,831	38,627	7,378	<0.001
	(43,870)	(45,289)	(25,229)	(46,680)	(51,534)	(40,263)	(42,711)	(59,425)	(47,302)	(10,725)	
MEDICAID											
Number of enrollees	585	52	93	29	70	7	62	68	381	204	
Total $	59,404	73,861	49,526	37,556	114,761	152,220	68,332	107,287	79,178	22,474	<0.001
	(74,919)	(79,958)	(76,721)	(50,914)	(108,040)	(126,234)	(57,119)	(59,904)	(82,735)		
Rx Drug $	5,254	4,612	4,063	4,936	12,224	19,478	4,306	6,159	6,401	3,113	<0.001
	(7,614)	(5,556)	(5,309)	(6,440)	(14,169)	(21,473)	(4,612)	(4,571)	(8,770)		
ER $	1,119	584	784	537	820	663	480	864	707	1,889	<0.001
	(11,530)	(1,021)	(1,491)	(1,045)	(1,389)	(855)	(748)	(1,697)	(1,318)		
Inpatient $	28,954	38,564	35,280	13,464	50,024	111,605	28,326	45,878	38,939	10,305	<0.001
	(62,084)	(71,797)	(72,032)	(38,892)	(101,144)	(122,921)	(51,091)	(54,231)	(72,752)		
Outpatient $	24,078	30,101	9,399	18,620	51,694	20,474	35,220	54,386	33,132	7,168	<0.001
	(28,650)	(20,080)	(7,624)	(20,389)	(35,506)	(13,681)	(24,433)	(36,987)	(31,159)		

A. Total known modalities is the sum of previous 7 columns (radiation through surgery + radiation + chemotherapy.

B. P-value is from chi square test for a significant difference between the treatment modalities (including unknown modality).

Adjusted to 2009$.

### Time trends in spending

**Figure 4:**
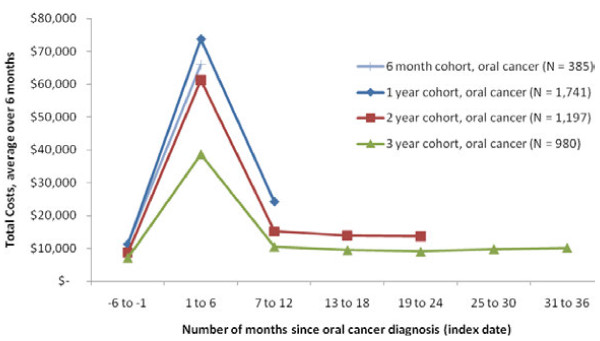
Trend in Total Healthcare Costs for Patients with OC/OP/SG Cancer, Commercial Coverage.

**Figure 5:**
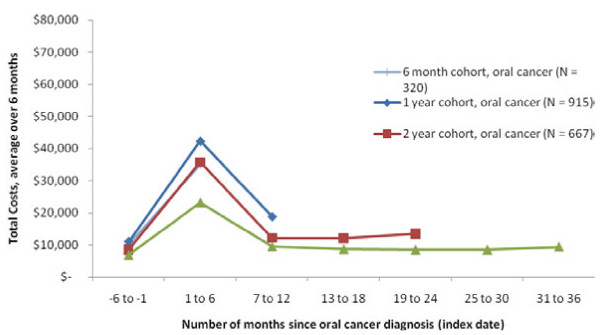
Trend in Total Healthcare Costs for Patients with OC/OP/SG Cancer, Medicare Coverage.

**Figure 6:**
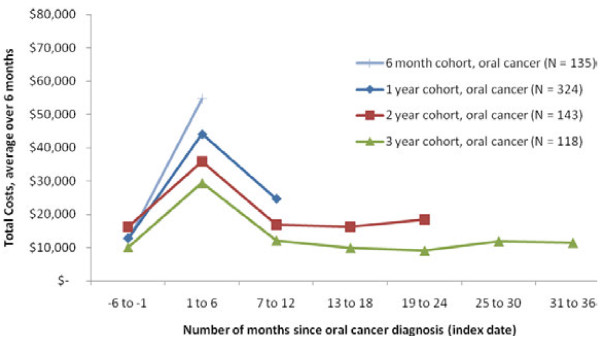
Trend in Total Healthcare Costs for Patients with OC/OP/SG Cancer, Medicaid Coverage.

As a secondary analysis, we calculated spending trends in 6-month intervals for all of the payer groups starting in the 6 months before diagnosis and continuing through three years, as enrollment allowed. Spending trends followed a similar pattern across all payer groups with lower spending in the 6 months preceding the index diagnosis date, followed by a spike in spending in the 6 months following diagnosis (Figures 4, 5, 6). After this spike, spending levels declined and stayed relatively stable in the next few years, but did not return to pre-diagnosis levels.

When analyzing time trends by cohort, in the Commercial and Medicare samples the cohort with at least 1 year but less than 24 months of post-index enrollment had the highest spending levels. For the Medicaid sample, the cohort with at least 6 months but less than 12 months of enrollment had the highest spending levels. As the cohort longevity increased (from 6 months to 3 years), costs tended to be lower.

### Short-term disability

Patients included in the indirect cost (short term disability) analysis were commercially insured employees (n = 281). The average number of disability days during the first year after the index diagnosis was 48.3 days, with most days incurred in the first six months after diagnosis. Indirect costs associated with STD were significantly higher for employees with OC/OP/SG cancer than employees without OC/OP/SG cancer -- patients with OC/OP/SG cancer had $17,876 in STD costs compared to $6,916 for the matched comparison group in the year after the index date (*p* < 0.01) (Table 5).

**Table 5:** Short Term Disability 1 year after index date for OC/OP/SG cancer patients and their matched comparison group among commercial enrollees

**VARIABLE**	**Patients with OC/OP/SG Cancer N = 281**		**Matched Comparison Group N = 281**		**Cost Burden**^**A**^	
	**COUNT/MEAN**	**%/STD DEV**	**COUNT/MEAN**	**%/STD DEV**	**Diff**	**P-value**^**B**^
Short-Term Disability (STD) Data						
Patients with any STD	125	44.5%	23	8.2%	36.3%	<0.0001
Number of days of STD among all eligible patients^C^	48.3	88.5	3.4	16.9	44.9	<0.0001
Number of days of STD among patients with any STD^D^	108.7	105.3	42.0	44.0	66.6	<0.0001
Cost among all eligible patients^E^	$7,952	$14,563	$566	$2,779	$7,386	<0.0001
Cost among patients with any STD^E^	$17,876	$17,322	$6,916	$7,240	$10,960	<0.0001

For sample with 1 year of follow-up.

Notes

A. Cost burden is the difference between mean costs for O/P cancer patients and their matched comparison group.

B. P-value from test of the significance of the difference between patients with O/P cancer and their matched comparison group.

C: All eligible patients are those found in the STD database. Patients may have had 0 days of STD.

D: Patients with any STD are all patients with at least one STD day.

E: STD payments were imputed by an hourly rate of $29.37, value each STD day at 70% compensation (e.g., STD Payment = STD days* 0.7*29.37*8).

## Discussion

The objective of this study was to determine the direct and indirect costs of OC/OP/SG cancer among cohorts of individuals in three payer groups: Commercially-insured enrollees and enrollees with Medicare and supplemental benefits from their employer, and a Medicaid population. Analysis of the Commercially-insured individuals revealed that the average medical costs of OC/OP/SG cancers in the first year after diagnosis was $79,151, which is significantly higher than the cost to treat other cancers ($31,559-$65,123) [26,27]. Furthermore, individuals who received surgery, radiation and chemotherapy averaged $153,892 during the year after diagnosis (Commercial sample). These medical costs are about twice any other reported cancer costs. These results are not surprising given the multiple modalities of treatment driven by the significant number of late or later stage diagnoses. What is unexpected is the magnitude of the total cost of cancer treatment. We found that the direct medical costs of OC/OP/SG cancer were substantially higher for all groups than the matched comparison group. For the commercial sample, total annual health care spending was $71,732 more than the comparison group while Medicare had total annual health care spending $35,890 higher than their matched comparison groups. Patients receiving both surgery and radiation treatment had the highest costs among the patients with OC/OP/SG cancer. For those individuals that survived the first year, indirect costs of short-term disability (STD) were also high and approximately double ($7,386 higher) for employees with OC/OP/SG cancer than for employees in the comparison group.

Further, it is not surprising to learn that the costs of care for cancer survivors did not return to baseline (before index date) after the first 6 months to 1 year. Post treatment cancer surveillance by physical examination and radiographic imaging is routine for several years after therapy is completed in order to detect recurrences early and when curative salvage therapy is still possible. Late effects from treatment are frequent and require ongoing management. Potential costs include physical and lymphedema therapy, swallow and voice therapy, dental care, nutritional support for those with a feeding tube, tracheostomy care expenses for those who have undergone laryngectomy, audiometric evaluation and treatment of hearing disorders resulting from treatment, and increased pharmacologic costs. Additional studies are needed to quantify the costs of surveillance and late treatment effects.

Our estimates of the expenditures for individuals with OC/OP/SG cancer are similar or higher than estimates reported in the literature. However, previous studies focused only on squamous cell carcinomas [3,5] and/or included the broader category of head and neck cancers [20,28,29]. The cost burden for patients with head and neck cancer has been observed to depend on patient and treatment related factors including: stage of disease at diagnosis, the treatment regimen, and the occurrence of high cost side effects [3,5,30]. While approximately 90% of patients with oral cancer have squamous cell cancer [20], our study was not limited to squamous cell carcinoma. By including salivary gland cancer, the average costs may have been lowered.

Our estimate of the cost of Commercially-insured individuals with OC/OP/SG cancer during the year after the index date ($79,151) was very similar to a previous study that found mean medical care costs of $80,070 (adjusted to 2009 USD$) for patients with squamous cell carcinoma of the oral/pharyngeal cavity who did not die in the first year after diagnosis (patients treated at a university hospital or clinic) [3]. Our estimate of Medicare costs for OC/OP/SG cancers ($48,410) was higher than found in previous studies ($22,321 [30] - $24,706 [29] in 2009$), although these studies examined the broader category of head and neck cancer.

There are two previous studies that report costs by treatment modality. The first, a Medicaid study [4] reported median costs of $27,020 for patients with treatment of presumed early-stage cancer, and $32,991 for patients with treatment of presumed late-stage cancer (2009 USD$). We found similar estimates for median costs in these data (medians not reported in the tables) that ranged from $28,820 (surgery only) to $74,579 (radiation only) for those with treatment.

The second study reported costs during the first 6 months after diagnoses for patients aged 35 and older with commercial insurance or Medicare with advanced squamous cell carcinoma of the head and neck [5]. The study reported $60,551 (radiotherapy alone) and $97,440 (chemoradiotherapy) total health care costs during the 6 months after diagnosis (all in 2009$). Six month costs in our study were comparable. For the Medicare sample, 6-month costs were $52,168 (N = 165) for patients with radiotherapy alone and $66,670 (N = 130) for chemoradiotherapy. In the Commercial sample, 6 month costs were $105,422 (N = 233) for radiotherapy alone and $137,315 (N = 361) for chemoradiotherapy.

It is worth noting that we found evidence of survivorship bias in this study; patients who were followed a longer period of time post-treatment had lower 6 month and annual costs. We cannot test survivorship directly since death is not well captured in administrative claims data (only in-hospital deaths can be measured). However, previous studies have reported a survivorship bias larger than that seen in our data; in one study patients who died in the first year after diagnosis had on average $50,000 higher costs than patients who did not die in the first year [3]. This suggests that some, but not all, of our loss to follow-up is due to death of a patient.

There are a number of limitations to these analyses. First, individuals with OC/OP/SG cancer were identified using ICD-9-CM diagnosis codes, which can be less precise than medical records or other clinical sources. When an individual did not have an inpatient claim with an OC/OP/SG cancer diagnosis, the patient was required to have at least two outpatient claims a minimum of 30 days apart, thus minimizing the inclusion of patients with “rule-out” diagnoses. However, it is not uncommon for the diagnostic process to take several months. The requirement for two outpatient claims outside a 30-day window could potentially result in a failure to capture cases.

We captured a large number of patients who did not have primary surgery or radiation. We examined the claims for this cohort and it appeared these patients received diagnosis-related care and not relevant surgery or radiation for OC/OP/SG cancer. Thus, the cohort may represent patients who underwent a lengthy and costly process to rule-out OC/OP/SG cancer and were eventually diagnosed as having benign disease. The inclusion of patients who may not have a cancer diagnosis may bias our estimate of the cost burden downward, creating a more conservative estimate. A second possibility is that our list of codes missed some treatments for OC/OP/SG cancer. It should be noted that a small share of patients without radiation or surgery did receive chemotherapy. This may represent a cohort of patients with metastatic disease who were treated with chemotherapy alone. It is also possible that a small number of patients may have had such late-stage cancer that hospice care was selected, rather than curative treatment. Finally, the radiation codes in our study are not site-specific and thus could represent radiation treatment on any part of the body.

While the indirect costs were substantial, our study used only short-term disability (STD) costs in estimating productivity burden, potentially resulting in an underestimation of these costs. We were not able to calculate indirect costs from absenteeism due to small sample size and did not capture information about lost productivity while at work due to OC/OP/SG cancer (presenteeism). We did not have estimates of the wages and benefits paid to employees on leave and valued a missed work day based on the typical benefits (70%) paid to the typical worker in the U.S. Patients with OC/OP/SG cancer tend to be older (average age in the Commercial sample was 53.4 years) than the typical worker. Older workers with more experience may be paid more than the typical U.S. worker, so our estimates of the short-term disability losses may be low. Patients with more severe OC/OP/SG cancer may have had to discontinue employment or may have been eligible for long-term disability (LTD) rather than STD, which would also add to the indirect costs. These indirect cost estimates must be interpreted with caution, given the small, non-random sample of patients with STD data. However, the limited data did reveal a significant cost burden to the employer. In fact, the average number of missed days of work as measured by days of STD in individuals with OC/OP/SG cancer (48.3 days) was greater than reports of all other cancers (44 days) [26] Side effects of cancer and its treatment cannot be over looked as important factors in managing working patients with cancer. A flexible workplace schedule is needed for those receiving radiation and/or chemotherapy. The side effects of fatigue, nausea, and vomiting may require the individual to limit the number of hours they can work.

Finally, the advantage of our matching approach is that we provide a reference for what the spending might have been for patients with OC/OP/SG if they did not have cancer, conditional on the characteristics used for matching. Thus we can account for the fact that OC/OP/SG cancer is not randomly distributed in the population. However, the ability to match is limited by what is observable in medical claims data. We believe our approach to selecting the reference group is conservative in the sense that it would lead to decreased cost differences between the OC/OP/SG cancer group and comparison groups because we match on health status characteristics at baseline as well as demographic characteristics. It is likely that we are finding a sicker and more costly comparison group than if we had simply matched on age and gender.

## Conclusion

This is the first retrospective data analysis of a large number of head and neck cancer patients in the U.S. analyzing direct and indirect costs, and comparing those costs to a matched comparison group., The overall cost burden of OC/OP/SG cancer is significant and is experienced by all payers, Medicare, Medicaid, the employer, and the individual. Both the direct costs and the indirect costs of OC/OP/SG cancer are high. In addition, patients treated with multiple modalities simultaneously are faced with some of the highest costs. The results of this analysis suggest that the treatment costs for OC/OP/SG may be the highest of all cancers in the U.S. Due to the fact that multi-modality treatment is more common for patients with late-stage OC/OP/SG cancer, early detection to find patients in earlier, less costly, stages of the disease is important. Having an estimate of the cost burden will help in determining the value of some of the new tools and improved methods available for early cancer detection that could be offered in employee medical and dental plans and/or employee wellness programs. Additionally, given the cost burden to both employer and employees, tobacco cessation programs and smoking bans should be reviewed. Lastly, the changing pattern of oral pharyngeal cancer dictates that further studies are needed to understand the influence of HPV on cancer in the workplace.

## Abbreviations

OC/OP/SG: Oral Cancer-oral pharyngeal cancer-salivary gland cancer; HPV: Human papilloma virus; CCAE: Commercial Claims and Encounters Database; COB: Coordination of Benefits; HIPAA: Health Insurance Portability and Accountability Act of 1996; IRB: Institutional Review Board; ICD-9-CM: International Classification of Disease 9th Revision, Clinical Modification; EPO/POS: Exclusive Provider Organization/Point of Service Plan; PPO: Preferred Provider Organization; HMO: Health Maintenance Organization; CCI: Charlson Comorbidity Index; PDGs: Psychiatric Diagnosis Groups; STD: Short-term Disability.

## Competing interests

Delta Dental of Michigan, Indiana and Ohio and Delta Dental of Wisconsin provided a grant to Thomson Reuters to support this research. Drs. Jacobson, Eichmiller, Carls, Gibson, Wang and Ms. Vogtmann completed this study as part of their employment at their respective organizations. Drs. Epstein and Murphy declare that they have no competing interests.

## Authors’ contributions

JJ contributed to the conception, design, analysis, interpretation of data and revising the manuscript. JB has been involved in conception, design, interpretation of data and revising the manuscript. FE has been involved in conception, design, interpretation of data and revising the manuscript. TB has been involved in conception, design, interpretation of data and revising the manuscript. GC has been involved in the design, analysis and drafting the manuscript. EV has been involved in the data compilation and design. SW has been involved in design and interpretation. BM has been involved in the interpretation of data, drafting and revising the manuscript. All authors read and approved the final manuscript.

## Supplementary Material

Additional file 1 HCPCS, CPT-4, and ICD-9-CM Codes for Identifying Surgical, Radiation and Chemotherapy Procedures. Click here for file
