# Supplementary material for: The cost burden of oral, oral pharyngeal, and salivary gland cancers in three groups: commercial insurance, medicare, and medicaid
Source: Head Neck Oncol. 2012 Apr 26;4:15. doi: 10.1186/1758-3284-4-15 (PMC3503554; doi:10.1186/1758-3284-4-15)
Supplement: Additional file 1 — HCPCS, CPT-4, and ICD-9-CM Codes for Identifying Surgical, Radiation and Chemotherapy Procedures. [file 1758-3284-4-15-S1.doc]

**Additional file** 1. HCPCS, CPT-4, and ICD-9-CM Codes for Identifying Surgical, Radiation and Chemotherapy Procedures

| **Surgery** |  |
| --- | --- |
| HCPCS | D7413, D7414, D7415, D7440, D7441 |
| CPT-4 | 11640-11646, 12001-14350, 15002-17315, 31365, 31368, 31390, 31395, 40800-40899  41000-41599, 41823, 41825, 41826, 41827, 41828, 41830, 41850, 42000-42299, 42300-42699, 42700-42999, 43020-43135, 60254, 69155 |
| ICD-9-CM | 6.4, 18.31, 24.31, 24.4, 24.5, 25.2, 25.3, 25.4, 27.31, 27.32, 27.42, 27.43, 27.49, 27.92, 28.2, 29.33, 29.39, 30.4, 40.40, 40.41, 40.42, 63.9 |
| **Radiation** |  |
| CPT-4 | 77261-77799 |
| ICD-9-CM | 92.2X,92.30, 92.31, 92.32, 92.33, 92.39, 92.41, 99.85 |
| **Chemotherapy** |  |
| CPT-4 | 96401, 96402, 96405, 96406, 96409, 96411, 96413, 96415-96417, 96420, 96422, 96423, 96425, 96440, 96445, 96450, 96521-96523, 96542, 96549, 99601, 99602 |
| ICD-9-CM | 99.25, 99.28 |
